# One Health: Animal Models of Heritable Human Bleeding Diseases

**DOI:** 10.3390/ani13010087

**Published:** 2022-12-26

**Authors:** W. Jean Dodds

**Affiliations:** Hemopet, 938 Stanford Street, Santa Monica, CA 90403, USA; info@hemopet.org

**Keywords:** animal models, human disease, heritable bleeding disorders, hemostasis

## Abstract

**Simple Summary:**

Animal models of human and animal diseases have been studied for decades in both experimental and clinical research with the findings applied to their management and therapy. Today, molecular and genomic research has led to the gene editing and gene therapies of an increasing number of these disorders. This review summarizes current knowledge about the molecular genetics and therapeutic approaches applied to the heritable human and animal bleeding diseases.

**Abstract:**

Animal models of human and animal diseases have long been used as the lynchpin of experimental and clinical research. With the discovery and implementation of novel molecular and nano-technologies, cellular research now has advanced to assessing signal transduction pathways, gene editing, and gene therapies. The contribution of heritable animal models to human and animal health as related to hemostasis is reviewed and updated with the advent of gene editing, recombinant and gene therapies.

## 1. Introduction

The identification of a group of mammalian genomes and their sequencing has led to the current genomic revolution (Table 1) [1]. In 2021, the Alliance for Regenerative Medicine published a review of the rare human diseases currently undergoing 61 different clinical trials which included examples of applied gene therapy [2]. A plethora of articles and opinions also has appeared in the recent global scientific literature [3,4,5,6,7,8,9,10,11]. These include hematological, ophthalmological and metabolic conditions. Most are based upon the CRISPR-Cas9 technology of Doudna and Charpentier and colleagues, along with the parallel work of Zhang and colleagues at Harvard and MIT’s Broad Institute [2,3,4,5,6,7,8,9,10,11,12].

As recently described and summarized by author Walter Issacson, the 2020 Nobel Prize winning genomic research of Jennifer Doudna and Emmanuelle Charpentier helped catapult molecular research into the CRISPR gene-editing era [12]. Similar findings were published by Virginijus Šikšnys and associates in Lithuania [12,13].

CRISPR is a genetic engineering technique that allows the genomes of living organisms to be modified primarily with a simplified bacterial CRISPR-Cas9 antiviral defense system that delivers the Cas9 nuclease complexed with a synthetic guide RNA (gRNA) into a cell. The genome of the cell can be cut at any desired location, such as Cas9 or Cas12, thereby permitting existing genes to be removed and/or new ones added in vivo [12,13,14,15].

Meanwhile, recombinant AAV vectors, based on nonpathogenic parvoviruses, have been used or are currently in use in 264 Phase I/II/III human clinical trials for these diseases [9,10,11,16,17,18,19,20,21,22,23]. While unexpected, remarkable clinical efficacy has also been achieved, the use of such high doses has been shown to provoke host immune responses culminating in serious adverse events including the deaths of four patients [9,10,16,17,18,19,20,21,22,23].

To address these limitations, scientists have developed capsid-modified next-generation (NextGen) AAV serotype vectors [9,16,17,18,19,20,21,22,23]. These new recombinant AAV vectors are up to 80-fold more efficacious at reduced doses. Regulatory approval using AAV5 gene therapy with valoctocogene roxaparvovec for hemophilia A was granted in the European Union in August 2022 and is pending in the United States, Meanwhile, the US FDA in November 2022 approved the AAV vector use of etranacogene dezaparvovec for treatment of adults with hemophilia B [16,24,25,26,27,28,29,30,31,32,33,34,35]. 

Additional data have derived from animal models to evaluate efficacy and safety, including the mouse, ‘humanized’ mouse, dog, monkey, and other non-human primates (Table 1). Viral vector characterization is also an important application in gene therapy [9,10,11,16,17,18,19,20,21,22,23,24].

Regardless, therapeutic gene and cell therapy research and development no doubt will progress when advanced technologies and services for viral vector design and manufacturing are adopted. Such technologies, in combination with technologies for CRISPR-based gene editing, RNA interference, base editing, and prime editing, will move innovative therapeutics forward [12,13,14,15,16,17,18,19,20,21,22,23,24,25,26,27,28,29,30,31,32,33,34,35].

This review addresses the topic with respect to the heritable mammalian bleeding disorders, the history of which is summarized below (Table 2). Please note that the acquired human bleeding disorders also are seen in animals (e.g., thrombocytopenia and thrombopathia, liver disease, rodenticide exposure and thrombosis with disseminated intravascular coagulation) [36,37].

## 2. Inherited Hemostatic Disorders in People and Animals 

**Figure 1:**
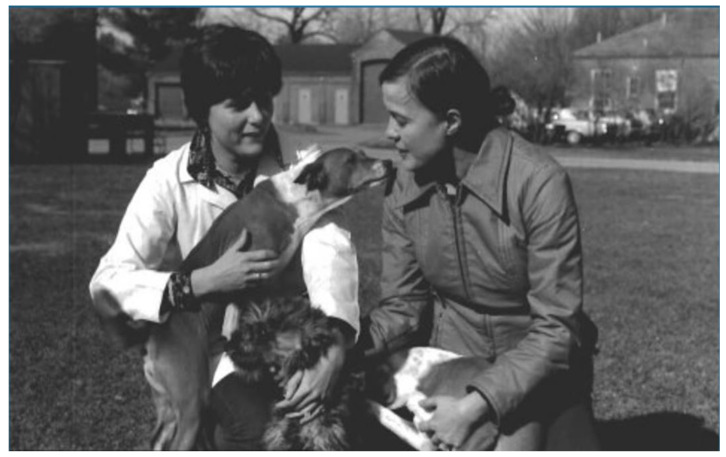
The Author and technician, the late Joanne Kull, with hemophilic dogs at Griffin Laboratory, New York State Department of Health, 1975 [39].

For decades, studies of the role of blood cells, plasma, lymph, and the vascular endothelium of humans have relied upon in vivo and in vitro animal models for their scientific advancements and understanding [19,22,25,29,31,36,37,38,39,40,41,42,43,44,45,46,47,48,49,50,51,52,53,54,55,56,57,58,59,60,61,62,63,64,65,66,67,68,69,70,71,72,73,74,75,76,77,78,79,80,81,82,83,84,85,86,87,88,89,90,91,92,93,94,95,96,97,98,99,100,101,102,103,104,105,106,107,108,109,110,111,112,113,114,115,116,117,118,119,120,121,122,123,124]. In that regard, hemostatic disorders that parallel those seen in people also have been recognized in companion animals for decades, and recent studies have focused on their management with recombinant and gene therapy [50,52,57] (Table 2 and Table 3). These heritable bleeding diseases occur most often as a consequence of inbreeding and line breeding—in rare breeds of dogs and cats, which by necessity are inbred, and in breeds in which particular animals are popular competition show winners and are used extensively for breeding [36,37,38,39,40,43,44,45,46,47,48,49] (Figure 1). Interestingly, the most common of all canine heritable disorders are essentially the same or similar in purebred and mixed breed dogs, as documented in a recent review that included more than 152 genetic disease variants in more than 100,000 dogs [84].

Diagnosis of these hemostatic disorders is more accurate when age-and sex-matched controls are used for coagulation studies [85,86,88,124], whereas for platelet function assessment. Mucosal bleeding time, whole blood, platelet-rich plasma and washed platelets have been the samples of choice [124] (Table 3).

The cloning of the factor VIII and factor IX genes occurred more than three decades ago [15,16,17,18,19,20,21,22,23,24,25,26,27,28,29,30,31,32,33,34,35,39,53,57,59,61,81] (Table 3). Since then, major advances in the application of molecular genetics and gene therapy to the diagnostics and clinical management of hemophilia have led to the generation of novel bioengineered recombinant clotting factor concentrates and the recent successes with AAV gene therapy for Factor VIII using AAV5, AAV6, AAV8, AAV-LK03, and AAVhum37 and factor IX with AAV-2, AAVS3L, AAV5, AAV6, AAV*, AAVSPK-100, and AAVrh10 ([16,17,18,19,20,21,22,23,24,25,26,31] (Table 1)).

Given these innovations and efforts, the clinical benefit of gene therapy in hemophilia has been relatively recent following on the outcome of Phase 3 clinical trials [25,30,31,34]. Despite the very high cost of development ($ 2.5 million for hemophilia A and $3.5 million for hemophilia B [34], these advances in gene therapy have clearly enhanced the safety and efficacy of hemophilia clinical care, reduced the described ‘societal burden’ from their care, and have vastly improved the quality of life of these patients [34]. 

The use of gene therapy has been and still is a major goal in hemostasis research ([16,17,18,19,20,21,22,23,24,25,26,27,28,29,30,31,32,33,34,35,39,59,61,62,124] (Table 2 and Table 3)). The first hemophilia gene therapy products approved for clinical use, as stated above, have used AAV gene therapy with the nonpathogenic parvoviruses, valoctocogene roxaparvovec for hemophilia A and etranacogene dezaparvovec for treatment of adults with hemophilia B [24,25,26,27,28,30,34]

A review by Kerri Wachter in the AABB News of February 2022, reviewed the gene biotherapy trials for patients with blood disorders [35]. Specifically mentioned was progress with sickle cell disease, and hemophilia A (Factor VIII deficiency) and B (Factor IX deficiency), which are caused by single-gene mutations [34,35]. These patients no longer should need prophylactic or therapeutic infusions of plasma or recombinant Factor VIII and IX, respectively. These gene therapies use adenovirus-associated viral vectors to deliver the missing DNAs to the patient’s liver for synthesis. This process of gene editing essentially deletes the mutant section of their DNA and inserts the normal section of DNA. In addition to gene editing. other genome approaches use gene addition, gene silencing and gene correction [5,6,7,8,9,10,11,12,35].

A severely affected young hemophiliac born since the mid-1990s with access to recombinant factor VIII and IX replacement therapy, can anticipate a normal life expectancy with little to no permanent complications from excessive bleeding [24,25,26,27,34,52]. This therapy when given by necessity every other day is exceedingly expensive, and there are still serious treatment concerns [30,34]. Firstly, some patients will develop neutralizing antibodies during the first 50 infusions of therapeutic factor VIII [30]. Secondly, placement of a central venous access device is typically needed which has the life-threatening risks of infection and thrombosis. Prolonging the biological efficacy of infused recombinant factor VIII has been a goal in this field [50,52,57].

Hemophilic boys receiving plasma-derived transfusion therapies in the early 1980s also had a 75–95% risk of acquiring human immuno-deficiency virus (HIV) and /or hepatitis C infection, respectively [34,50]. Since 1990, however, improved screening of plasma donors and commercial plasma fractionation and production protocols have shown no transmission of HIV, hepatitis C, or other virus associated with any of these modern plasma-derived factor VIII preparations [27,28,34,35,50].

The availability of several commercial recombinant factor VIII, factor IX, and von Willebrand factor products since the mid-1990s has largely supplanted the plasma-derived products [30,34,50,52,57]. When recombinant von Willebrand protein is infused along with recombinant factor VIII, the risk of developing a clotting factor inhibitor is reduced. Similarly, the amount of human albumin that the cell lines need for stability in producing the recombinant factor VIII has been reduced in each step, making the product safer [50,52,57].

Currently, a dozen or more gene transfer, gene editing and genetically modified cell therapy trials for hemophilia have been performed and are ongoing [16,17,18,19,20,21,22,23,24,25,26,27,28,29,30,31,32,33,34,35].

### 2.1. Hemophilia A (Factor VIII: C Deficiency)

Hemophilia A is an X-chromosome-linked recessive disease carried by the female and manifested in the male. In animals, female hemophiliacs can be produced, however, by the mating of hemophilic males to carrier females [36,37]. This situation has occurred with mild forms of the disease in inbred families of purebred dogs and cats where affected males survive to sexual maturity and can reproduce. Hemophilia is the most commonly reported, severe inherited coagulation defect of animals, and has been recognized in most breeds of dogs, in mongrel dogs, in many breeds of cats and mixed breed cats, in horses and cattle [36,37,39,40,41,42,43,44,45,46] (Table 3).

Recombinant human factor VIII produced by Genetech, Inc., was first infused in early 1970 into a Boxer dog with hemophilia A in this author’s clinic in rural Albany, NY [28]. The small volume of concentrated factor VIII took about five minutes and the toenail bleeding time checked beforehand at over ten minutes was dramatically reduced to just 3 drops of serous-tinged fluid. Thus, this breakthrough study in a hemophilic dogs showed clinical success of a commercial recombinant human factor VIII [27,36,37,38,50,51,52]. Parallel infusion and gene therapy studies in hemophilic dogs included not only plasma-derived and recombinant factor VIII but also recombinant factor VIIa to induce its sustained expression, as therapy to help generate factor Xa and bypass the need for factor VIII [29,57,59,61,62,66].

### 2.2. Hemophilia B (Factor IX Deficiency; Christmas Disease)

An X-chromosomal-linked recessive disease like hemophilia A, hemophilia B has been reported in at least 26 breeds of dogs and 3 breeds of cats [36,37,38,39,40,41,42,43,44,45,46,47,48,49,51] (Table 3). It was first recognized in families of the Cairn Terrier and British Shorthair cat [36,37,40,43,44,45]. Results of diagnostic screening tests are basically the same as those as described for hemophilia A although specific tests are required to identify the defect factor IX deficiency rather than factor VIII:C deficiency. Affected animals have very low circulating levels of factor IX and carrier females have levels reduced to about half normal (40–60%) [36,37,45].

Treatment and management considerations for pets with hemophilia B are the same as those for hemophilia A except that canine factor IX-rich plasma fractions are given [36,37]. In general, cats with either form of hemophilia are more easily managed as house pets than are dogs with the same diseases. Cats tend to more agile and are lighter and can often lead reasonably healthy, long lives maintained as house pets [40]. Recombinant human factor VIIa and IX infusions and gene therapy have also have been successful with canine hemophilia B [37,51,57,59,61,62,67].

### 2.3. von Willebrand Disease (vWD)

The multifaceted syndrome known as vWD was first described in humans in 1926 [38]. The first animal model of this disease was described in 1959 in a colony of Poland-China swine, and in 1970, the canine form was discovered in members of a German Shepherd Dog family imported from Germany [39,64,65](Table 3). Their disorder, usually milder than the hemophilias, causes bleeding from mucous membranes and skin, as well as epistaxis, and gastrointestinal and urogenital bleeding. Affected dogs have a prolonged bleeding time with resulting abnormal hemorrhage from surgery. While vWD is the most common inherited bleeding disorder of humans, it also occurs in about five dozen canine breed types, several families of cats, a quarter horse, and an inbred line of Flemish Giant/Chinchilla laboratory rabbits [47,64,65,66,67,68,69,70,71,72,73,74,75,76,77,78,79,80,81,82,83,84]. A high prevalence of the gene for vWD is found in the Doberman Pinscher (~80% prevalence), German Shepherd Dog, Miniature Schnauzer, Golden Retriever, Shetland Sheepdog, Basset Hound, Standard Poodle, Keeshond, Rottweiler, Dachshund, Scottish Terrier, Manchester Terrier, and Pembroke Welsh Corgi. The disorder is either less prevalent or the true prevalence is unknown in other breed types likely because too few animals have been studied [64,65,66,67,68,69,70,71,72,73,74,75,76,77,78,79,80,81,82,83,84].

Type 1 vWD is by far the most common form in canines, and is inherited as an autosomal, incompletely dominant trait with variable clinical and laboratory expression depending upon the degree of penetrance of the mutant gene [36,37,76,77,78,79,80,81,82,83,84]. vWD is analogous to the autosomal recessive Type 3 vWD of humans in four dog breeds: Scottish terriers, Chesapeake Bay retrievers, Shetland sheepdogs, and German wirehaired pointers, in Himalayan cats, and Poland-China swine [35,68,71,73,76]. Homozygous affected individuals cannot produce measurable von Willebrand factor (vWF) and have a moderate to severe bleeding tendency. vWD heterozygotes can be detected by laboratory tests as they have reduced vWF antigen or activity, but are otherwise asymptomatic.

Many additional, less common variants of vWD exist and are classified as Type 2 vWD. These include families of German shorthair pointer dogs, and quarter horses [36,70,82]. Concurrent hypothyroidism can exacerbate bleeding in canine vWD, resulting in the situation where asymptomatic carriers of vWD may exhibit a bleeding tendency if they develop autoimmune thyroiditis and become hypothyroid. This is a common situation that is especially prevalent in Doberman pinschers [36,72]. Hypothyroid dogs also may exhibit thrombocytopenia and mucosal surface bleeding (Table 3). As thyroid supplement non-specifically shortens the bleeding time in animals with mild inherited or acquired vWD and other platelet dysfunctions, clinical experience with its use supports the efficacy, safety and low cost of this approach [36,37,72].

A reliable genetic screening test for identifying Scottish terriers with type 3 vWD is available for this and several other breeds [76]. Strong associations were detected between plasma von Willebrand factor concentration and von Willebrand factor marker genotype. All were homozygous for a 157-base pair intragenic marker allele and homozygous or compound heterozygous for 1 of 4 extragenic marker alleles. These marker genotypes were exclusively detected in dogs with low plasma von Willebrand factor concentration, although some dogs with these genotypes did not have abnormal bleeding [76].

### 2.4. Inherited Platelet Function Defects

Inherited disorders of platelet function were originally characterized in humans as either being of the Glanzmann’s thrombasthenia or Bernard-Soulier syndrome types. Since then, a wide variety of heritable and acquired platelet disorders called thrombopathias have been identified in people and animals [36,37,39,101,102,103,104,105,106,107,108,109,110,111,112,113,114,115,116,117,118,119,120,121,122,123,124,125] (Table 3). Thrombasthenia is an autosomal disorder where both sexes are affected and both sexes can carry the gene for this disease. In animals, it was first recognized in a family of Otterhound dogs bred by a veterinarian in upstate New York [36,37,101,108,114]. A similar disease has been recognized in other hound breeds and in an occasional cat. The biochemical defect on the membrane or surface of affected animals′ platelets is similar to that of human Glanzmann’s disease, and is caused by a deficiency of platelet GPIIb/IIIa which results in reduced platelet aggregation [113,114]. A deficiency of GPIB-IX-V in Bernard-Soulier Syndrome cases a platelet adhesion defect [106,123]. In the otterhound disorder, the platelets are large as well as dysfunctional; a similar disorder with large “Swiss-cheese”- like platelets was identified in a human patient [101,102]. 

Basically, the clinical signs of these disorders are similar to those of vWD because patients have long bleeding times. Platelet numbers are usually normal in these diseases but the function of the platelets is impaired. In the thrombopathic disorders, affected animals are born with defective platelet function. These are of several biochemical types and have been recognized in Basset Hounds, American Foxhounds, Spitz, Greater Swiss Mountain Dogs, German Shepherd Dogs, Simmental cattle, several breeds of cats and in a family of Fawn Hooded rats (strain named FH/Wjd) ([103,111,112,113,115,116,117,118,119,120,121,122,123,124] (Table 3)). The clinical signs, again, are similar to those of vWD because the animals have long bleeding times. The disease in Basset Hounds has been quite widespread among North American breeding stock and is caused by an unique, platelet activation signaling defect problem following injury to a blood vessel [106,108,109]. More recently, other platelet disorders have been described in animals, including ADP storage pool deficiency, ADP receptor defect, signaling pathway defects (CalDAG-DEFI and Kindlin-3) for GPIIb/IIIa activation, and a platelet procoagulant defect (Scott Syndrome) resulting in an in vivo coagulopathy [104,117]. A recent in-depth review summarized these findings [6,24] (Table 3).

### 2.5. Other Inherited Disorders

#### 2.5.1. Factor VII Deficiency

Factor VII deficiency is a mild to moderate bleeding disorder in people characterized by bruising, and soft tissue bleeding from gums, bowel and urinary tract [37,47,89]. It was described in the 1960s in colonies of Beagles bred for biomedical research [37]. Since then, dogs affected by this autosomal recessive trait have been useful for studies that require monitoring liver function, as factor VII is synthesized in the liver and has a very short half-life (~4 h). A novel missense mutation has been identified as causing the relatively high prevalence of this defect in the breed [89].

#### 2.5.2. Factor X Deficiency

Stuart–Prower factor (factor X) deficiency, an uncommon human coagulation disorder, was first described in the 1970s in a family of American Cocker Spaniels [90]. This condition has since been diagnosed in mongrel dogs and the Jack Russell terrier. Very low levels of factor X (<6% to 35%) are present in homozygotes and some heterozygotes, and they have a clinically expressed bleeding disease, whereas most heterozygotes (40–70% factor X) are asymptomatic. When factor X activity is below 20%, the affected dogs usually do not survive neonatal life. Severely affected pups are stillborn or fade and die in the first week or two of life, thereby mimicking the ”fading puppy syndrome”. Necropsy of these pups reveals massive internal bleeding. Signs in adults are mild and bleeding is seen from mucosal surfaces [37,90,91].

#### 2.5.3. Factor XI (PTA) Deficiency

Another rare disorder of humans, factor XI deficiency mostly affects individuals of Jewish background [37,38]. Spontaneous bleeding episodes are mild (hematuria, bruising, epistaxis, menorrhagia) except when patient undergoes surgical procedures. In this case, bleeding usually starts 12–24 h after surgery and can be severe and protracted. Even after minor procedures such as biopsies and tonsillectomy, lethal bleeding has been reported. In animals, this disorder is clinically like the human equivalent and was first described in English Springer Spaniels. It also has been reported in Holstein cattle, Great Pyrenees, and Kerry Blue Terrier dogs [37,92,93,94,95].

#### 2.5.4. Prekallikrein (Fletcher Factor) Deficiency

Prekallikrein is involved in the early surface contact phases of blood clotting. It is the precursor of plasma kallikrein that activates small peptide kinins. In addition to humans, deficiency of prekallikrein has been reported in a family of Belgian horses, and two dog breeds [98,99,100]. In one affected dog, a point mutation was identified in Exon 8 leading to an amino acid substitution in the fourth apple domain of the protein [99].

#### 2.5.5. Factor XII Deficiency (Hageman Trait)

An asymptomatic coagulation deficiency recognized in humans, Hageman Trait (factor XII deficiency) occurs in dogs, and is quite often found in cats [37,96,97,100]. The absence of detectable biological or immunological factor XII is a normal phenomenon of a variety of other species, such as whales, birds (including the common domestic fowl and waterfowl), reptiles, and possibly fish [37].

## 3. Discussion and Conclusions

Research on animal models has been pivotal essential to our understanding of basic and applied sciences and has led to significant improvements in the management of both human and animal diseases [36,37,38,39,40] (Table 2 and Table 3). Veterinarians and animal scientists have been at the forefront of biomedical research in comparative medicine over the last 50 years [38,39]. The study of naturally occurring or induced animal models of human disease has led to tremendous growth of knowledge in many disciplines, including hematology, immunology, vaccinology, virology and genetics and contributed significantly to new areas of research, such as transplantation and gene therapy [34,36,37,38,39,40,55].

This era began with in vitro manual diagnosis using tilt tube timed assays with test tubes and a 37 °C water bath along with skin and mucosal surface bleeding times [39]. Today, sophisticated genetic, genomic and molecular diagnostics plus the use of safe, blood type compatible blood transfusion products, including blood concentrates, recombinant and stem cell technology are available for humans and companion animals [36,37]. As described above, the first early study with a recombinant human clotting Factor VIII product was infused into a hemophilic boxer in our laboratory at the Griffin Laboratory, NYS Department of Health in Albany, NY. His bleeding time normalized for 48 h and this success help lead to human clinical trials with this technology [27,28,29,30,31,32,33,34,35].

These research animal models also benefitted other animals [37,39,48,49,88,110,116,124]. While information generated from animal-based research experiments has been used primarily to benefit human health and well-being, parallel benefits have been accorded to animals. A classical example is the inherited bleeding disorders discussed here. In fact, this author was surprised how relatively easy it was over the last decades to search for and find parallel animal models of the human diseases of interest [36,37]. The net effect of those basic and comparative medical advances has been to improve diagnostic and treatment modalities in clinical veterinary medicine [36,37,39,48,49].

## 4. Conclusions

Current molecular markers and gene editing research has yielded practical and innovative clinical applications. For decades, veterinary and comparative geneticists have developed and relied upon biochemical markers of specific genetic traits to identify carrier and affected animals that are used as models of human disease [39,85,86,87,88,124]. More recently, molecular approaches have been developed that can be used to study gene therapeutic approaches for advancing human and animal health and well-being [1,2,3,4,5,6,7,8,9,10,11,12,13,14,15,16,17,18,19,20,21,22,23,24,25,26,27,28,29,30,31,32,33,34,35,50,51,52,53,88,124], Future technological developments, particularly in the areas of gene delivery and cell transplantation, will be critical for the successful clinical implementation of this gene therapy.

## Figures and Tables

**Table 1:** Timeline of animal genome sequencing [1].

Species	Date Sequence Published
Human	2001
Mouse	2002
Rat	2004
Chicken	2004
Non-Human Primate	
Chimpanzee	2005
Rhesus macaque	2007
Orangutan	2011
Dog	2005
Cat	2007
Cow	2009
Horse	2009
Turkey	2010
Pig	2012
Goat	2017

**Table 2:** Scientific history of blood coagulation [36,37].

Time Period	Discoveries
1700s	Long after Hippocrates, Aristotle, Celsus and Galen found freshly drawn blood to clot, blood clotting became linked to hemostasis (the cessation of bleeding).
1800s	Thrombosis first recognized by Virchow; platelets are discovered by Bizzozero; familial bleeding tendency in males (hemophilia) is first recognized.
1900s	Morawitz described the classical theory of blood coagulation.
1930–1940s	Disputes arose between scientists about factors that form and dissolve clots; more clotting factor disorders are recognized in people (von Willebrand Disease) and dogs (hemophilia).
1950s	von Willebrand disease identified in Poland-China pigs; factor VII identified in dog plasma after coumarin therapy prolonged the blood clotting in vitro.
1970–1980s	von Willebrand Disease described in German Shepherd Dogs imported to North America from Germany, and then in many dog breeds, cats, and rabbits; hemophilia described in cats and horses; factors I, IX, X, XI and XII deficiencies documented in dogs, cats, cattle, goats; and platelet defects described in dogs, rats, and mice.
1990s–today	More of these bleeding disorders found in domestic and companion animals, including the documentation of familial pre-kallikrein and kallikrein deficiencies.

**Table 3:** Genome Wide Associations (GWAS) for heritable canine bleeding disorder traits.

Bleeding Disorder	GWAS; Genes	Breeds Affected	References
Hemophilia A (Factor VIII Deficiency)	Boxer, single nucleotide change C to G at nucleotide 1412 (1412 C>G)in Exon 10, results in arginine to proline at amino acid 471 (P471R) in A2 domain German Shepherd Dog, single nucleotide change G to A at nucleotide 1643 (1643 G>A)in Exon 11, results in tyrosine to cysteine at amino acid 548 (C548Y) in A2 domain	Many, also mixed breeds, cats, horses	[19,21,29,39,43,56]
Hemophilia B (Christmas Disease; Factor IX Deficiency)	Missense mutation G to nucleotide 1477, glycine 379-glutamic acid Insertional mutation in line 1 of canine FIX gene Nucleoside deletion of transcription factor binding site of FIX gene promotor	Cairn Terrier, Hovawart, German Wired-Haired Pointer (Drathaar), 23 other breeds, and cats	[16,20,33,58,59,60,61,62,63]
von Willebrand Disease, Types 1, 2, 3	Type 1, Doberman, homozygous 157-base-pair intragenic marker allele+ heterozygous 1 of 4 extragenic marker alleles Type 2, GSHP nucleotide variant at Exon 28 Type 3, single nucleotide deletion in Exon coding VWF prepeptide (Scottish Terrier), splice site mutation Intron 16 (Dutch Kooiker)VWFc.4937A>G A/A, G/G	Many, prevalent in Doberman Pinscher, Shetland Sheepdog, Scottish Terrier, Golden Retriever, Pembroke Welsh Corgi, Chesapeake Bay Retriever, German Short-Haired Pointer (GSHP), German Wire-Haired Pointer (Drathaar), ~ 50 other breeds, cats, Poland. China swine	[76,77,78,81,82,83,84,88]
Factor VII Deficiency	Missense G96E mutation at Exon 5. Glycine 26 to Glutamic acid, 31% frequency in breed	Beagle, more than 14 other breeds	[89]
Factor X Deficiency (Stuart–Prower Disease)	Homozygous deletion of factor X gene(s) is lethal	American Cocker Spaniel, Jack Russell Terrier	[91]
Factor XI Deficiency	Kerry Blue, mutation of F11 gene, homozygotes affected, 90 bp insertion, Chr16:44477343-44477344, 10 bp duplication (dup GCACAAAGCT) Chr:44477344-44477353	English Springer Spaniel, Kerry Blue Terrier, and Holstein cattle	[95]
Factor XII Deficiency (Hageman Trait)	Cats, novel mutation (c.1631 G >C) at Exon 13 of feline F12 gene, results in amino acid change (p.GS54A)	Miniature Poodle, cats, reptiles, marine mammals, birds	[96,97]
Prekallikrein Deficiency	G to A transversion at Exon 8	Shih Tzu. American Hairless Terrier, others, and Belgian horse	[99]
Thrombasthenia (Glanzmann’s Disease); Bernard-Soulier Syndrome	Otterhounds, single nucleotide change at G1193 (1000) at Exon 12 of gene encoding for glycoprotein GPIIb, substitution of histidine for aspartic acid at 398 (367) of calcium -binding domain of GPIIb Single ITGA2B gene mutation on chromosome 9, chr9:19054488-19054488: G>C American Cockers, single glycoprotein 9 (GP9) deletion at Exon coding on chromosome 20 Great Pyrenees, 14-base insertion in Exon 13 and a splicing defect of Intron 13 Deletion of P2Y12 in Greater Swiss Mountain Dog and Bichon Frise	Otterhounds American Cocker Spaniel, Greater Swiss Mountain Dog (GSMD), Great Pyrenees, Bichon Frise	[113,114,121,123,124]
Thrombopathia	RASGRP-1; chr18:52417313-52417315: 3 bp deletion (del TCT) Autosomal recessive procoagulant deficiency at canine chromosome 27	Basset Hound, Spitz, and cats, Simmental cattle, Greater Swiss Mountain Dog, German Shepherd Dog, Fawn-Hooded (FHwjd) rat	[103,106,108,111,112,115,117,121,122,124,125]
Thrombocytopenia	Associated with Hashimoto’s lymphocytic thyroiditis (3-5 genes of major histocompatibility complex, MHC, as in humans)	American Cocker Spaniel, Old English Sheepdog, Standard Poodle, Vizsla, Weimaraner, Akita, Samoyed, Shih Tzu, Long -Haired Dachshund, Kerry Blue Terrier, other white/fawn and dilute-color breeds and hybrids	[118,120]
Macrothrombocytopenia		Norfolk Terrier, Cairn Terrier, Chihuahua, Danish-Swedish Farm Dog, Kritikos Lagonikos, Wesr Highland White Terrier, Parson Russell Terrier, Marenma and Abruzees Sheepdog	[124]

## Data Availability

Data for these studies can be found in the cited literature.
